# Cortico-striatal activity associated with fidget spinner use: an fMRI study

**DOI:** 10.1038/s41598-023-43109-7

**Published:** 2023-09-22

**Authors:** Suzuka Narukawa, Momoka Nishimura, Izumi Kuze, Ibuki Ohno, Masaki Fukunaga, Kohta I. Kobayasi, Shota A. Murai

**Affiliations:** 1 Faculty of Life and Medical Sciences, Doshisha University, Kyotanabe, Kyoto 610-0321 Japan; 2 Division of Cerebral Integration, National Institute for Physiological Sciences (NIPS), Okazaki, Aichi 444-8585 Japan; 3 Department of Physiological Sciences, School of Life Science, SOKENDAI (The Graduate University for Advanced Studies), Hayama, Kanagawa 240-0193 Japan; 4 Graduate School of Life and Medical Sciences, Doshisha University, Kyotanabe, Kyoto 610-0321 Japan; 5 International Research Center for Neurointelligence (WPI-IRCN), The University of Tokyo Institutes for Advanced Study, Hongo, Tokyo, 113-0033 Japan

**Keywords:** Psychology, Human behaviour, Neuroscience, Motor control, Sensorimotor processing

## Abstract

Fidget spinners are said to be a very successful toy, and it's said that it has a good impact on attention for children with ADHD and hand motor control. However, there is limited scientific evidence to support these claims, and there is a lack of data on neurobiological responses to rotating fidget spinners. To better understand the mechanism whereby fidget spinners affect motor behavior, we tried to identify the neural correlates of rotating fidget spinners using functional magnetic resonance imaging and non-magnetic fidget spinners with five types of ease of rotation. As a result, we confirmed that the pre/postcentral gyrus, middle temporal gyrus, supplementary motor area (SMA), cerebellum, and striatum are activated when rotating spinners. Furthermore, the SMA was activated more with easier-to-rotate spinners. Additionally, a psychophysiological interaction analysis revealed increased functional connectivity between the SMA and the caudate while rotating fidget spinners compared to just holding them. These results suggest that the fine motor control associate with spinning a fidget spinner is supported by the cortico-striatal circuits involved in planning and reward.

## Introduction

Fidget spinners are toys, played by rotating them, and producing a vibration. People were so enthusiastic about playing with fidget spinners that students were not allowed to bring them to some elementary school classes due to their constant use^1,2^. The fidget spinners' spin was said to have a positive effect on humans. Particularly, the motto “*Fidget spinners enhance attention in ADHD individuals*” was used for its promotion. However, although some researchers suggest that fidget spinner use by individuals with attention deficit and hyperactivity disorder (ADHD) improves attention^3^, others claim the opposite^4,5^. In addition, it is said that fidget spinners can improve hand motor control^6–8^. However, despite these claims, it is yet unclear how fidget spinners affect human behavior.

The impact on humans using fidget spinners is attributed to the act of fidgeting with them. Fidgeting is the action of moving one's own body or another object that is not essential for the current event^9^ and is an impulsive behavior seen in individuals with ADHD^10,11^. For example, a fidgeting body may improve motor control^12,13^. In the context of fidget spinners, “fidgeting” consists of a series of flicking, gripping, and the experience of fine vibration. Particularly, Cohen and colleagues suggested that fidget spinners improve precise hand movements^6^. In their research, some participants either rotated a fidget spinner, held it, or did nothing. Thereafter, the participants performed a spiral-tracing task, and their precision was evaluated. As a result, participants who rotated or held a fidget spinner traced spirals more precisely, increasing their fine motor control. Although fidget spinners are not the only approach to enhancing hand motor control, they may be effective as they are enjoyable and simple to use. To investigate whether fidget spinners affect motor behavior, the neural system underlying spinning fidget spinners needs to be investigated. Previous functional near-infrared spectroscopy (fNIRS) studies that measured brain activity during behavioral tasks after rotating a fidget spinner were conducted to investigate whether brain activity during fine motor tasks changed after spinning a fidget spinner^7,8^. Healthy adult participants were divided into two groups: one group spun a fidget spinner before fine motor tasks, while the other did not spin a fidget spinner^7^. Results showed no difference in task performance between groups. However, during the fine motor tasks that were highly cognitively loaded, the group that spun a fidget spinner exhibited a decrease in ΔHbO (change in oxygenated hemoglobin) levels in the dorsolateral prefrontal cortex (DLPFC). Additionally, the authors also conducted the study using the same protocol in adults with ADHD^8^. The ADHD participants also showed improvements in motor tasks after using the fidget spinner. These studies showing beneficial effects of fidget spinners suggested that fidget spinners may help people focus their attention on hand movement and consequently improve fine motor control. However, to our knowledge, no study has focused on measuring brain activity during rotating fidget spinners to provide insights into the potential neural mechanisms of such fidgeting that attract people and enhance motor behavior.

**Figure 1:**
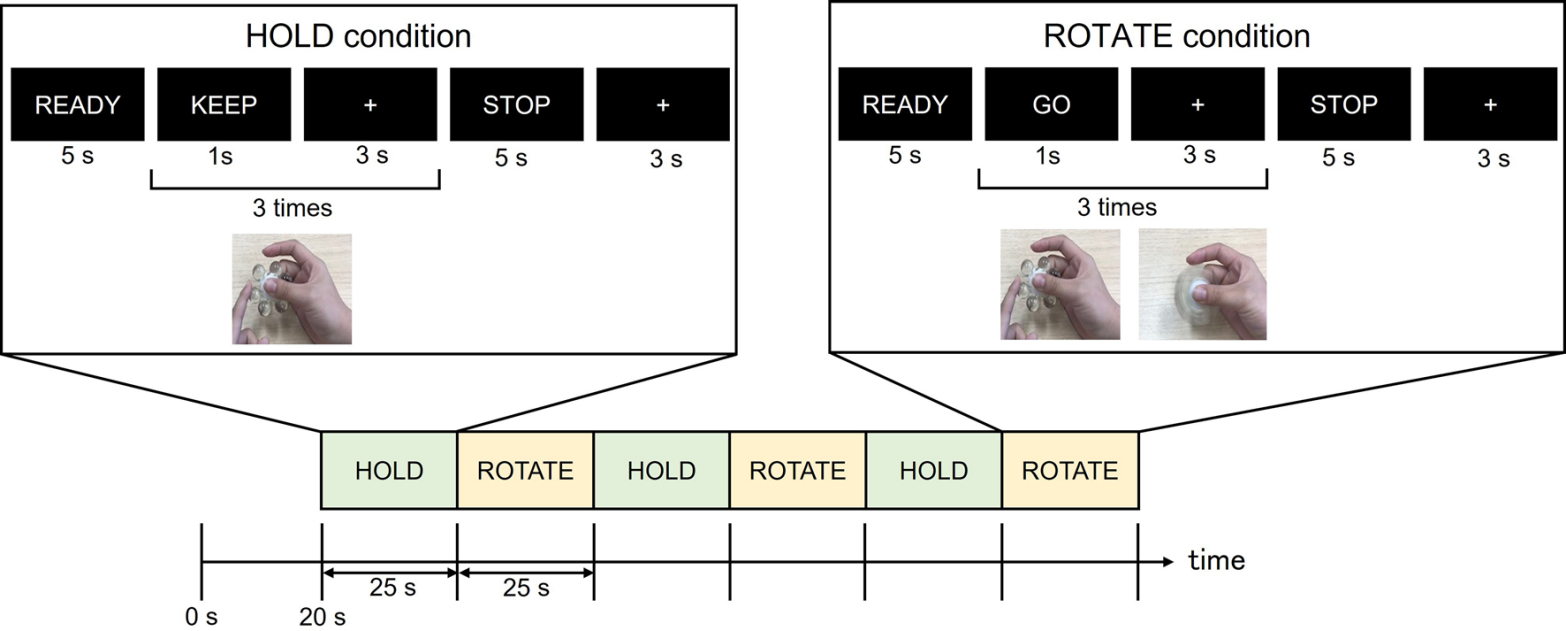
Procedure for the MRI task. When "READY" was displayed, participants got ready to rotate the spinner. They held the spinner in their right hand and were ready to flick it with their left hand. In the ROTATE condition, participants rotated the spinner when "GO" was displayed on the screen and stopped the spinner when "STOP" was displayed. In the HOLD condition, participants only held the fidget spinner and did not rotate it.

In this study, we aimed to identify the brain mechanisms associated with rotating fidget spinners. The major attraction of fidget spinners may be caused by their ease of rotation (eccentricity) and low friction. Given the relationship between the attraction and the ease of rotation, we assume that a core region involved in fidgeting using a fidget spinner is sensitive to its ease of rotation. We therefore developed non-magnetic fidget spinners of various eccentricities, asked participants to rotate and hold the fidget spinners in a magnetic resonance (MR) scanner (Fig. 1) and explored brain regions showing differential activity for the ease of rotation. Ease of rotation might affect motor planning while rotating fidget spinners. The supplementary motor area (SMA) is a higher-level motor area^14–19^, linked to motor planning^20,21^, preparation^22,23^, and suggested to be involved in precision grip^19,24^. Thus, our first hypothesis is that the SMA is differently activated at different eccentricities. In addition, given the attractiveness induced by fidgeting behavior with spinners^1,2^, the motor process for such a fidgeting behavior may be involved in reward processing, and the fidgeting behavior may bias attention towards hand movement and consequently improve fine motor control^6,7^. This possibility is supported by studies showing that activation of the ventral striatum, a part of basal ganglia and the dopamine circuit^25,26^, is related to the reinforcement of movements that result in a reward^27^. These make our second hypothesis that rotating fidget spinners increased functional connectivity between the brain region showing activation associated with the ease of rotation and the ventral striatum.

## Results

### Ease of rotating fidget spinners

**Figure 2:**
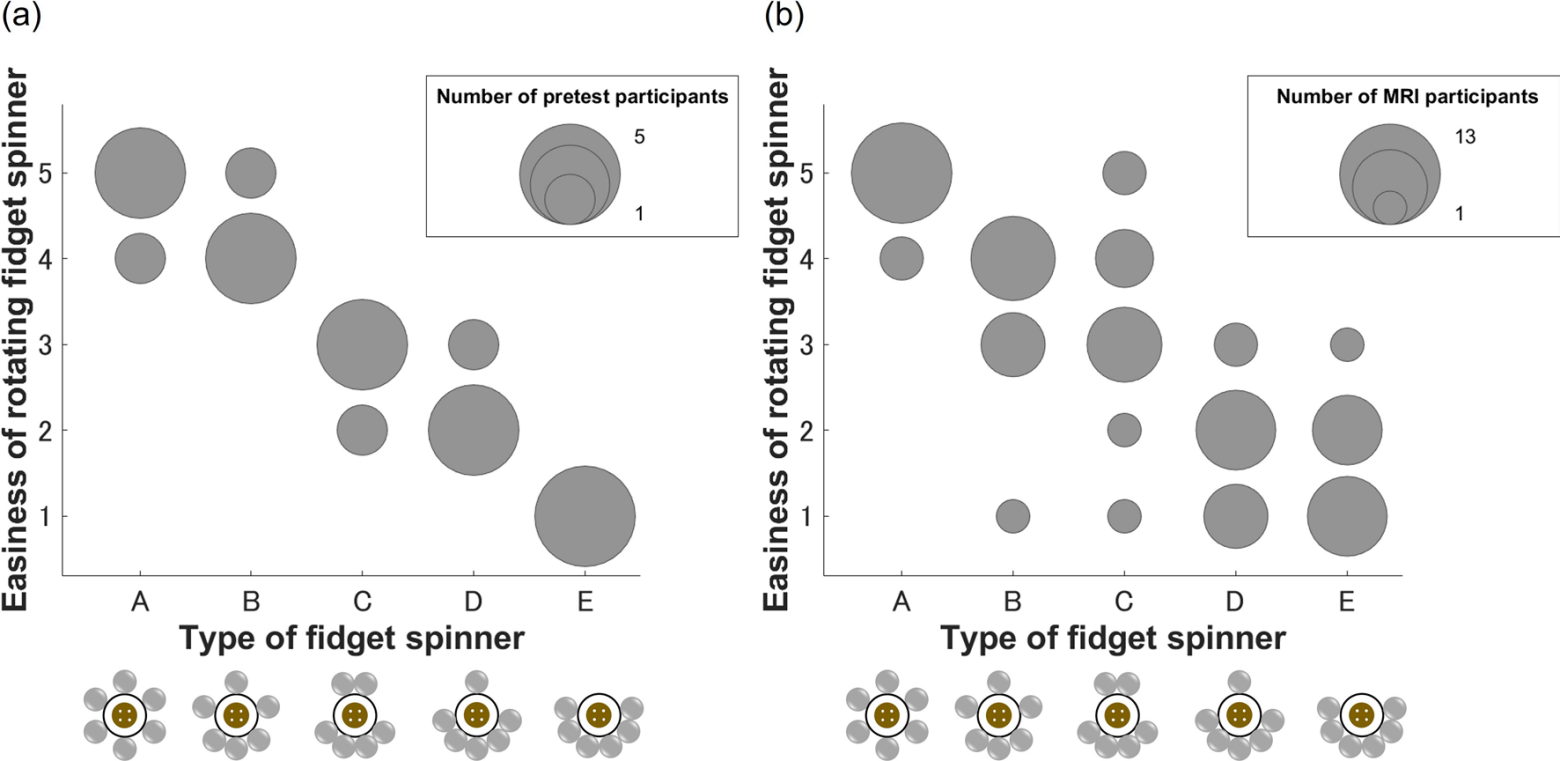
Ease of rotation for each type of fidget spinner. The participants evaluated ease of rotation of the fidget spinner. (Left) Participants who did not participate in the fMRI task rated the ease of rotation. (Right) Participants who participated in the fMRI task rated the ease of rotation in the MR scanner.

First, we investigated the relationship between eccentricity and the ease of spinning a fidget spinner. We determined the ease of spinning in a naturally seated state as well as lying down, considering the participants’ atypical position for using a fidget spinner in the MRI scanner. The ease of spinning was evaluated by the participants solely based on the sensation of spinning, without observing the shape of the fidget spinner. In the pretest, participants who did not join the main experiment evaluated the ease of rotation of five-type fidget spinners in a naturally seated state (Fig. 2 left). The order of the subjective ease of spinning and that of small eccentricity were the same. Similarly, participants who joined the fMRI task were asked to rate the ease of rotation in a lying down (Fig. 2 right). After the fMRI task, participants were instructed to rotate the fidget spinners while lying in the MR scanner and to report ease of rotation for each fidget spinner. Fidget spinners were rated in the same order of eccentricity as in the pretest.

### Brain activity observed during fidget spinner rotation

**Figure 3:**
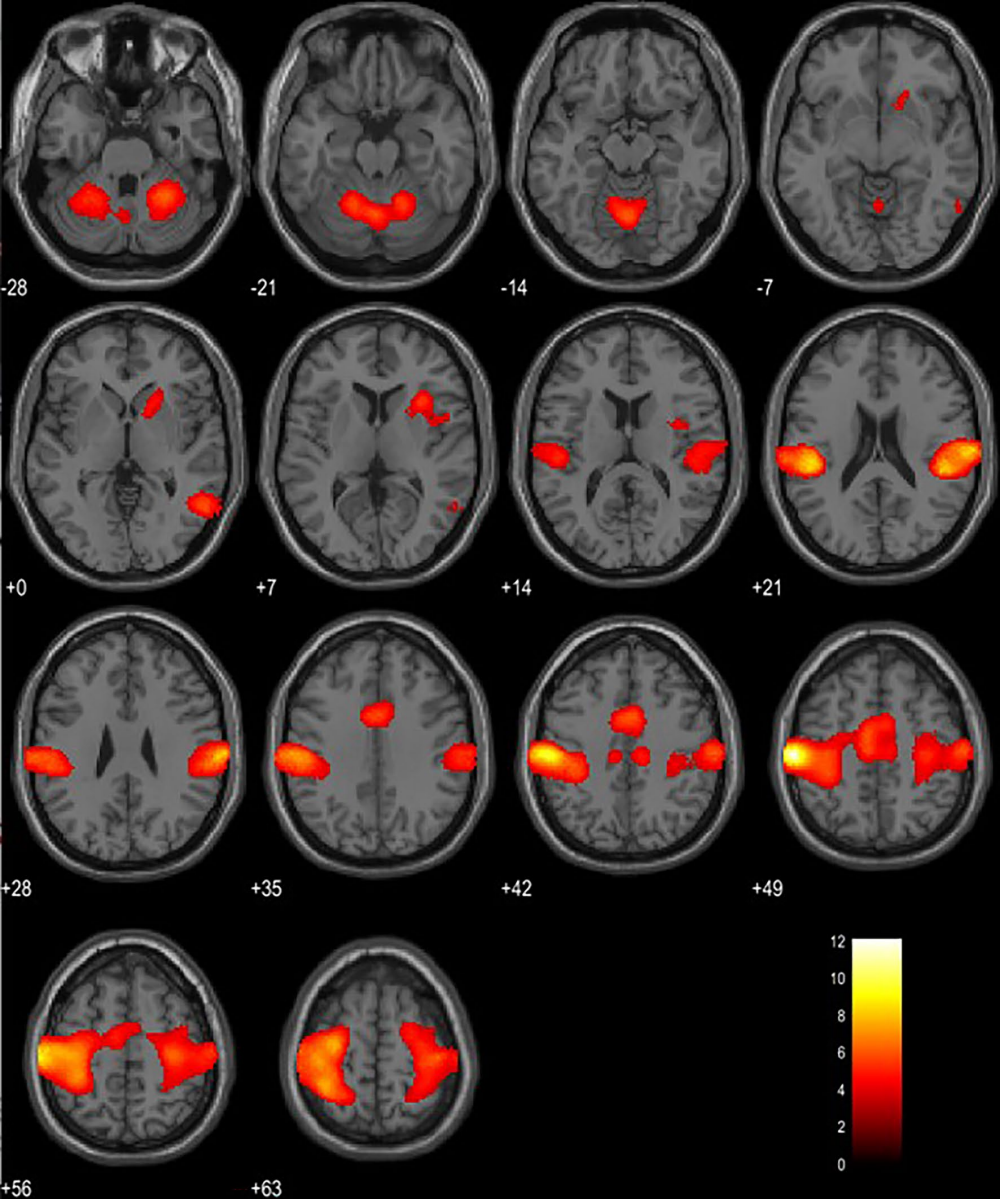
Brain activity when rotating spinners compared to just holding spinners (N = 22). The height threshold was set at *p* < 0.001 (uncorrected for multiple comparisons) and the cluster threshold was set at familywise error (FWE) corrected p < 0.05. L, left hemisphere; R, right hemisphere; z = MNI coordinates. The template was a single T1 subject in SPM.

We measured brain activity while rotating a fidget spinner (ROTATE condition) and while simply holding a fidget spinner (HOLD condition). A direct comparison of the differences between the ROTATE and the HOLD conditions revealed stronger activation in the inferior parietal lobule, pre/postcentral gyrus, paracentral gyrus, supplementary motor area (SMA), supramarginal gyrus, middle temporal gyrus, vermis, cerebellum, insula, and putamen (see Fig. 3, Table 1).

**Table 1:** Clusters of brain regions showing significant activation.

Spatial extent test		MNI coordinates			T-value	Hemisphere	Anatomical region
cluster size (mm^2^)	P-value	x	y	z			
ROTATE > HOLD							
87,224	< 0.001	− 60	− 22	50	12.11	L	Inferior parietal lobe
		− 48	− 26	20	8.76	L	Rolandic operculum
		− 32	− 12	70	8.70	L	Precentral gyrus
58,576	< 0.001	66	− 22	26	8.50	R	Supramarginal gyrus
		48	− 30	20	8.06	R	Rolandic operculum
		60	− 30	24	6.80	R	Supramarginal gyrus
3368	< 0.05	58	− 58	2	6.40	R	Middle temporal gyrus
		68	− 54	0	4.16	R	Middle temporal gyrus
21,320	< 0.001	− 2	− 64	− 16	6.34	L	Vermis
		30	− 52	− 28	6.34	R	Cerebellum
		24	− 58	− 24	6.27	R	Cerebellum
5016	< 0.01	32	16	8	5.64	R	Insula
		20	16	− 2	5.25	R	Putamen
		40	0	12	4.54	R	Insula
Ease of rotation							
2744	< 0.01	14	− 8	58	4.88	R	Supplementary motor area
		− 4	− 8	60	4.28	L	Supplementary motor area
		4	− 10	58	4.13	R	Supplementary motor area
PPI analysis							
224	< 0.05*	6	12	− 2	4.52	R	Caudate

The local maxima separated by more than 8 mm per cluster were shown up to three. Regions were labeled using automated anatomical labeling (AAL3). x, y, and z = Montreal Neurological Institute (MNI) coordinates in the left–right, anterior–posterior, and inferior-superior dimensions, respectively. n = 22. *p* < 0.05 cluster-level FWE corrected.

*In the PPI analysis, the statistical threshold was peak level FWE corrected to p < 0.05 with small volume correction within the ventral striatum.

**Figure 4:**
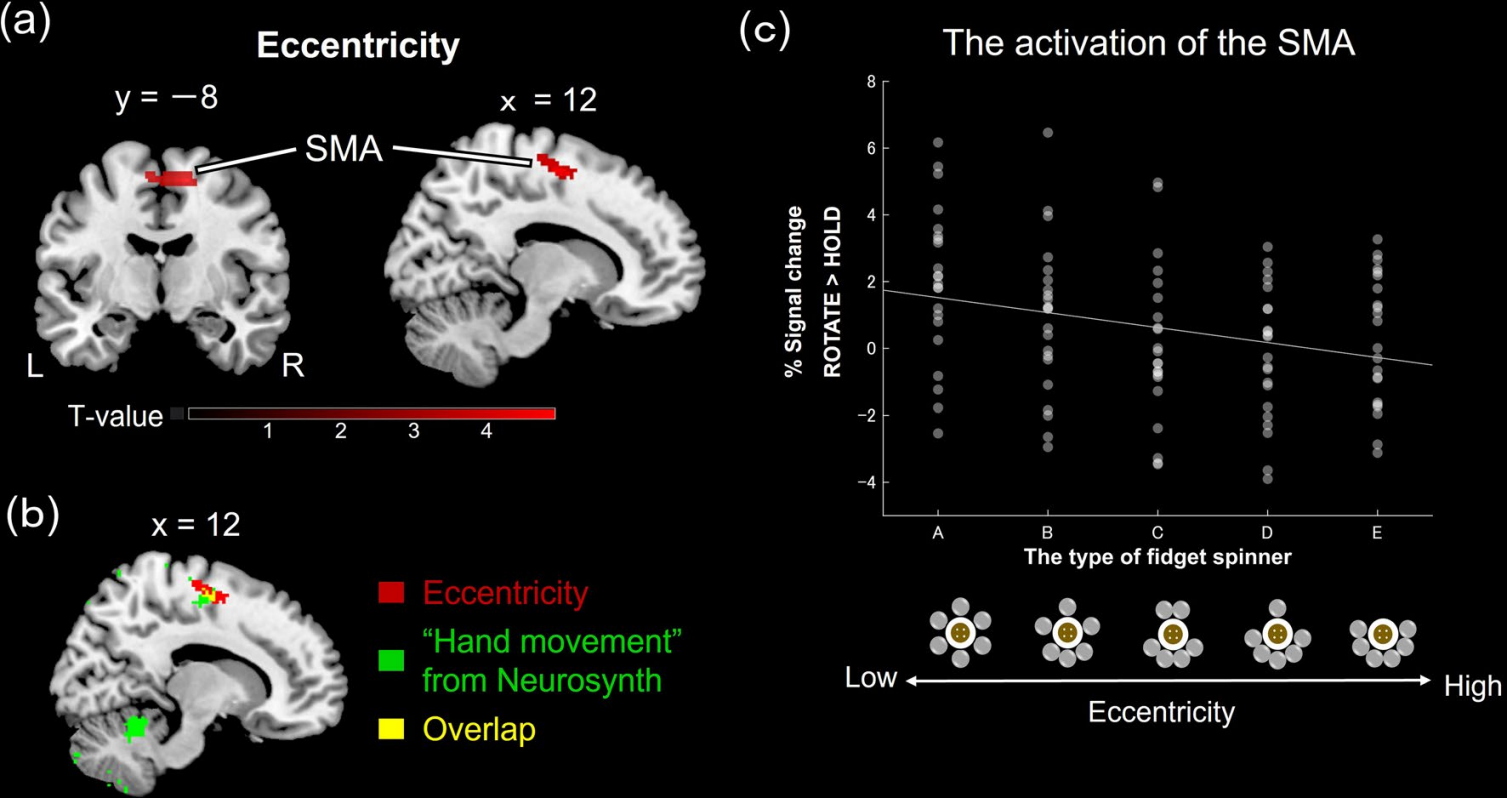
Brain activity associated with eccentricity. (**a**) BOLD signal changes (ROTATE > HOLD) in the supplementary motor area (SMA) decreased with a changing degree of eccentricity. The height threshold was set at *p* < 0.001 uncorrected for multiple comparisons and the cluster threshold was set at FWE corrected *p* < 0.05. (**b**) The overlapping between the "hand movement" regions searched by Neurosynth and the SMA which was related to eccentricity. (**c**) Visualization of the relationship between brain activity and types of fidget spinners. Plots indicate individual data.

We also conducted a whole-brain analysis to examine the graded activity from spinner A, with low eccentricity, to spinner E, with high eccentricity. The lower eccentricity was associated with increased activity of the SMA (Fig. 4; Table 1). The regions of this SMA overlapped with the regions corresponding to hand movements reported in previous studies that were searched using Neurosynth (https://neurosynth.org/). We also collected a survey showing self-reported usage of fidget spinners in minutes per month [mean = 19.7 min per week, range = 0–150 min per week]. We then applied the usage time of spinners as a covariate to the fMRI group analysis and tested the effect of familiarity on activation for ROTATE compared to that for HOLD. The result did not show the effect of usage time (cluster level FWE corrected p > 0.05).

**Figure 5:**
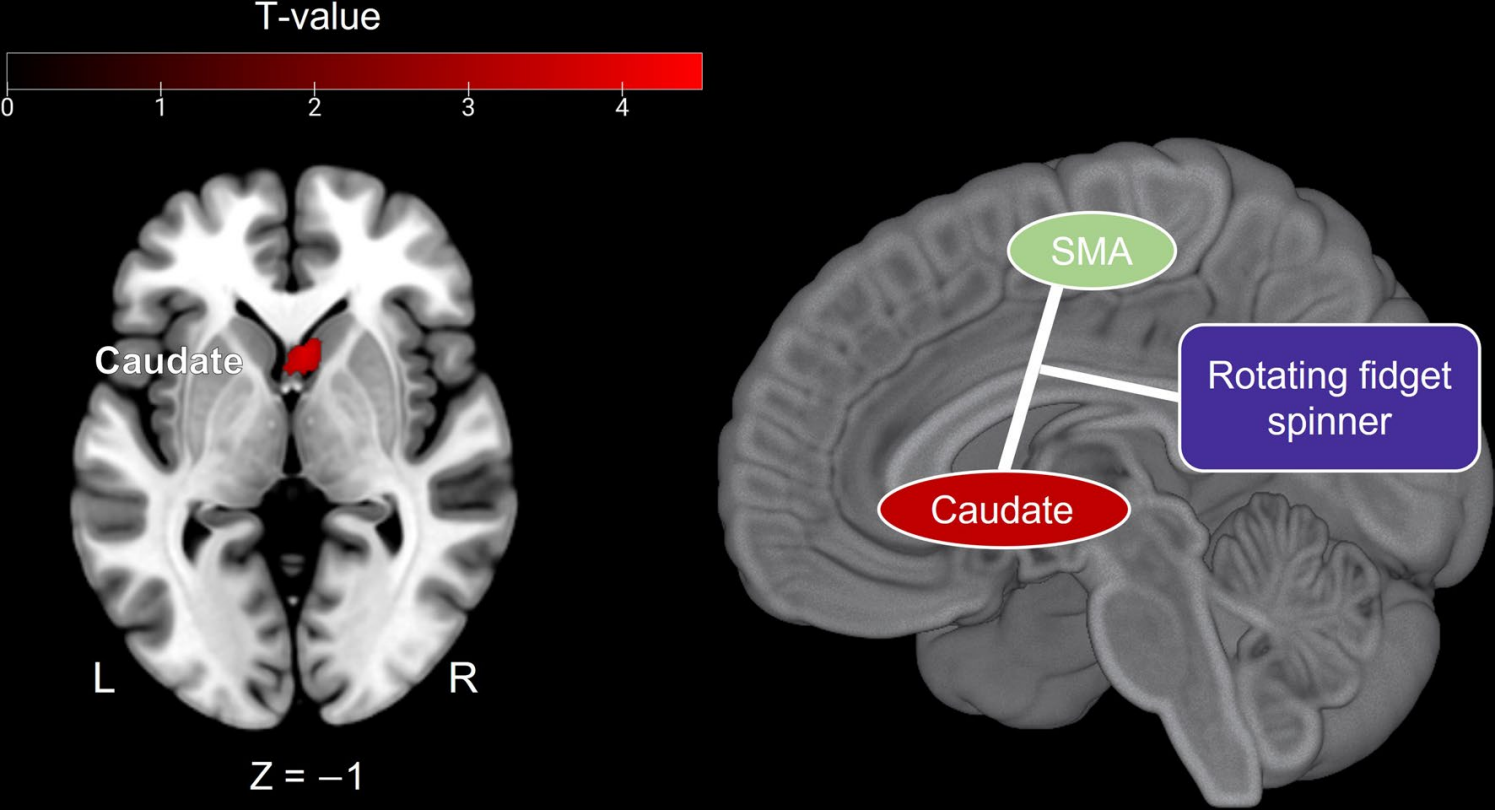
Physio-physiological interaction (PPI) analysis with the seed regions in the SMA. We found increased connectivity between the SMA and the caudate under the ROTATE condition compared to the HOLD condition. The statistical threshold was set at peak level FWE corrected *p* < 0.05 with small volume correction using anatomical masks (i.e., ventral striatum). SMA, supplementary motor area.

Finally, we conducted a psychophysiological interaction (PPI) analysis to examine whether the SMA (Fig. 4) related to eccentricity was linked to the ventral striatum (caudate, nucleus accumbens) for fine motor control. Drawing an 8-mm-radius sphere ROI centered at the SMA peak that responded to eccentricity (x = 14; y =  − 8; z = 58) as a seed region for the PPI analysis, we observed significantly increased coupling between the SMA and the caudate in the ROTATE condition versus HOLD condition (Fig. 5).

## Discussion

We conducted an fMRI study to examine brain activity while rotating fidget spinners in an MR scanner. We created a non-magnetic fidget spinner with different degrees of ease of rotation by controlling eccentricity levels. While rotating the spinners, the pre/postcentral gyrus, middle temporal gyrus, supplementary motor area, cerebellum, and striatum were more activated than when just holding the fidget spinners. We also investigated the effect of eccentricity on brain activity. Rotating a fidget spinner with lower eccentricity led to greater activity in the SMA. In addition, rotating fidget spinners increased the connectivity between the SMA and the caudate.

In this study, we confirmed that rotating fidget spinners activated sensory and motor regions like the pre/postcentral gyrus, the middle temporal gyrus, the supplementary motor area, the cerebellum, and the striatum. These activations were observed in previous precise grip studies^28,29^, and might have been elicited by flicking, gripping, and sensing the fine vibration occurring while rotating the spinners. In addition, the activation seen was mostly bilateral. Participants flicked the fidget spinner with their left hand while holding it with their right hand. That way is a typical method to rotate a fidget spinner, and easy even for those who have never used a fidget spinner. It is likely that both hands were involved in fine motor control, leading to bilateral activity observed.

While rotating fidget spinners with low eccentricity, activated more the SMA, indicating that SMA activity is related to the ease of rotation. We offer two possible explanations for these results. First, preparation of movement might be more facilitated with fidget spinners of lower eccentricity. The SMA has been associated with movement preparation phase^21–23^,and the process is involved in motor planning and prediction^21,22^. All participants had previously played with other fidget spinners as they practiced the task before entering the MR scanner. Therefore, the short-term experience using fidget spinners might allow participants to build the process of preparation to rotate the fidget spinner by integrating relatively simple somatomotor inputs. However, when using a fidget spinner with high eccentricity, the process for movement preparation might not be easily adjusted due to complexity of the movement. Second, the fidget spinner with low eccentricity that produces small vibrations requires finer hand movements to hold it. The SMA has been suggested to affect not only overall motor planning and execution but also fine motor control^24^ and related to inhibition of unnecessary motion^22^. Lower eccentricity might require more subtle hand movements, leading to increased SMA activity. The Neurosynth meta-analysis localizer showed the brain regions for hand movement, and the regions overlapped with the peak coordinate of the SMA activation we found. This suggests that the regions where activity varies depending on eccentricity may be related to the motor planning of hand movements in the SMA.

Furthermore, according to the PPI analysis, functional connectivity between the SMA and caudate increased in the ROTATE condition compared to the HOLD condition. In other words, when rotating a fidget spinner, the SMA and caudate activation were closely linked. The connectivity between the SMA and the striatum serves to adjust fine movements^30,31^. In addition, given that the caudate is a part of the dopamine circuit, it is plausible that rewarding the fidgeting behavior can occur during rotating fidget spinners. Consequently, this fidgeting-induced reward might bias attention towards handling a hand, leading to a sustained impact on both movement and attention immediately following the rotation of the fidget spinner.

The previous fNIRS study^7^ investigated brain activity while performing complex fine-motor control tasks after spinning a fidget spinner. The results demonstrated a decrease in ΔHbO in the DLPFC after spinning a spinner. The activation during fidget spinner rotation could be related to the subsequent motor task; however, the current study solely focused on the neural correlates of spinning a spinner and did not examine its impact on fine motor control followed by fidget spinner rotation. To better understand the potential mechanisms that affect motor behavior, continuous measurement before and after using a spinner will be necessary.

Additionally, although we did not compare a difference in brain activation between fidgeting and non-fidgeting movement, we examined activation differences in eccentricity enabled us to identify the specific motor regions involved in the ease of rotating fidget spinners, linking with the attraction of fidgeting. This motor region furthermore exhibited enhanced functional connectivity with the reward circuits encompassing the ventral striatum during the rotation of the fidget spinner. The ventral striatum is associated with ADHD symptoms like impulsive behavior and shows hypoactivation during reward anticipation in ADHD^32^. A recent study conducted by Koiler et al. demonstrated improvement in fine motor control in adults with ADHD following the use of a hand spinner^8^. The SMA-ventral striatum circuitry might be involved in the beneficial effect on ADHD individuals, although further study is needed to investigate whether and how fidget spinners affect the ventral striatum activity specifically in ADHD individuals.

In conclusion, this is the first study to investigate the whole-brain neural activity associated with fidget spinners, which have been commercially successful, though with little scientific support. We found that neural activity in the SMA depends on the ease of rotating fidget spinners and that the fidget spinners’ attraction might be associated with the cortico-striatal circuit. These findings shed new light on the neural mechanisms underlying fidgeting with this gadget.

### Limitations

This study investigated the relationship between rotating fidget spinners and brain activity and does not necessarily endorse the use of fidget spinners. Future research will be required to directly examine the biological mechanisms behind the potential benefits of rotating fidget spinners, such as improved attention, to clarify the impacts of spinners more comprehensively. In addition, it is unclear whether the caudate activity observed in this study is linked to the dopaminergic system. To examine the relationship between dopamine and spinning a fidget spinner, further research will need to use molecular imaging techniques such as positron emission tomography.

## Materials and methods

### Fidget spinners

**Figure 6:**
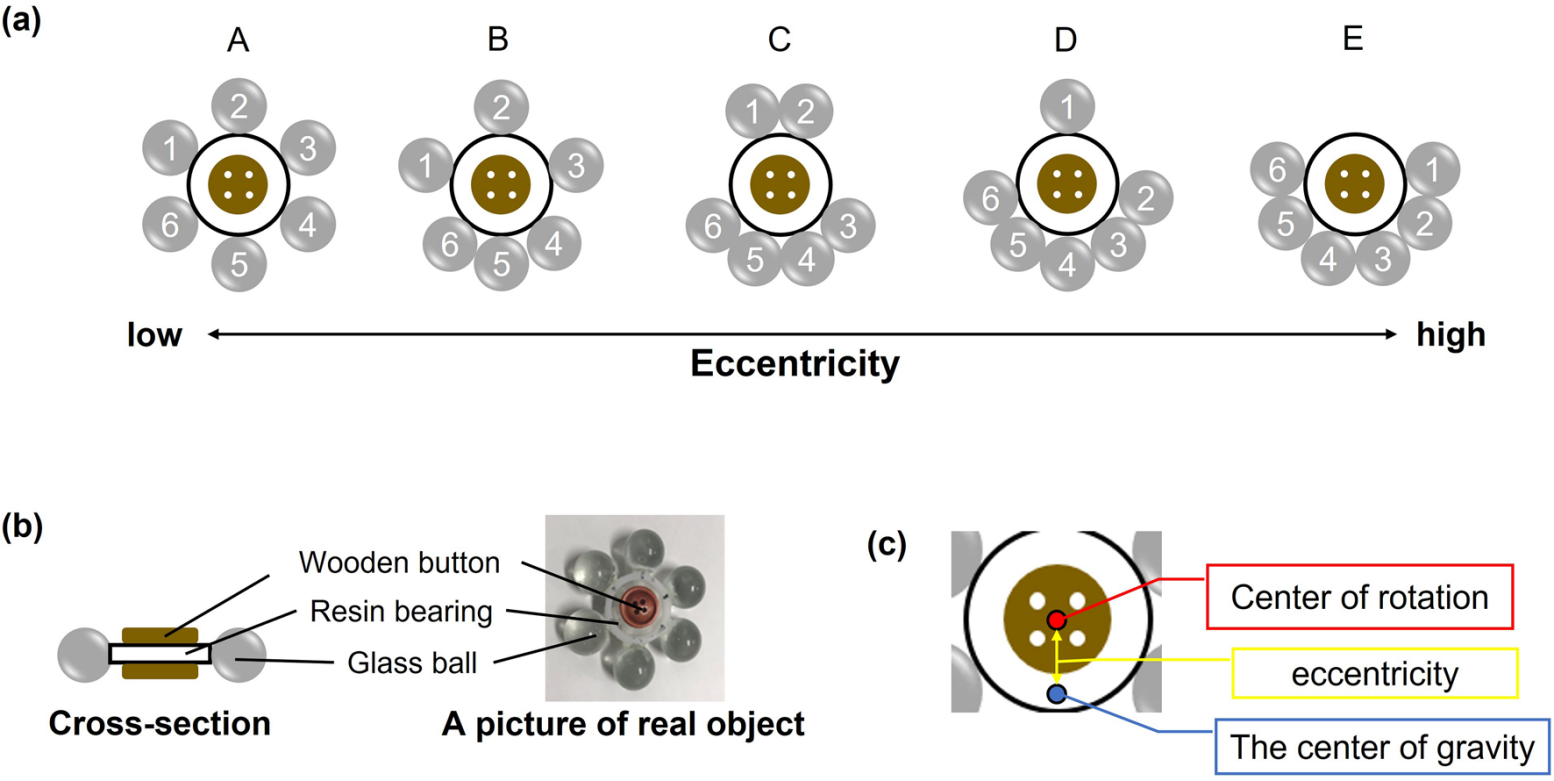
Non-magnetic fidget spinners with variable degrees of ease of rotation. (**a**) Five types of spinners with different eccentricities. The numbers written on the glass balls are identification numbers to calculate eccentricity. (**b**) Details of fidget spinner construction. (**c**) Eccentricity indicating distance between the center of rotation and center of gravity.

Non-magnetic MRI-compatible fidget spinners (Fig. 6a) with variable degrees of ease of rotation were constructed and used for the whole experiment. They consisted of a resin-made ball bearing (PE-26-PHP10, TOK, Inc.) and six glass balls (Fig. 6b), with wooden buttons attached to both sides of a bearing as finger-holding places by two kinds of adhesive (Aron Alpha EXTRA, Toagosei Co., and Super X Hyper wide, CEMEDINE Co., Ltd.). The five types of fidget spinners varied depending on the glass ball placement. "A" was the fidget spinner with the lowest eccentricity while "E" was the one with the highest eccentricity (Fig. 6a,c; Table 2).

**Table 2:** Properties of the fidget spinners.

Type of fidget spinner	Weight of glass ball (g)						Angles between number-1 ball and other glass balls (°)						Eccentricity
	1	2	3	4	5	6	1	2	3	4	5	6	
A	6.11	6.14	6.14	6.15	6.11	6.18	0.00	60.0	120	180	240	300	0.27 × 10–3
B	6.11	6.11	6.12	6.11	6.11	6.11	0.00	70.0	140	205	250	295	0.12
C	6.10	6.10	6.10	6.10	6.10	6.10	0.00	45.0	135	180	225	270	0.13
D	6.12	6.12	6.12	6.12	6.12	6.12	0.00	88.0	130	180	228	272	0.21
E	5.98	5.98	5.95	6.02	5.94	6.09	0.00	45.0	91.0	138	184	230	0.28

Of each fidget spinners, glass balls’ weight, angles between the ball No.1 (see Fig. 6a) and the other balls, and eccentricity.

Then, we calculated the eccentricity of each fidget spinner. The position of the center of bearing and each ball could be written using $(x,y)$ coordinates. The distance from the center of the bearing to the center of each glass ball was set to 1 for convenience; thus, the center of bearing and ball no. 1 can be put $(x,y)=(\mathrm{0,0})$ and $\left(\mathrm{1,0}\right),$ respectively. Using each glass ball’s weight (*m*) (Fig. 6a; Table 2) and the angles between the reference ball and each ball ($\theta$; Fig. 6a; Table 2), the position of center of gravity was obtained by the formula below.

1 $$\left(x,y\right) = \left(\frac{{\sum }_{i = 1}^{6}{m}_{i}cos{\theta }_{i}}{{\sum }_{i = 1}^{6}{{m}_{i}}},\frac{{\sum }_{i = 1}^{6}{m}_{i}sin{\theta }_{i}}{{\sum }_{i = 1}^{6}{{m}_{i}}}\right)$$

Finally, the eccentricity was calculated as the distance from the center of rotation to the center of gravity.

### Pretest

A preliminary rating test (pretest) was counted to confirm the relationship between eccentricity and ease of rotation. Five healthy young adults (two females; ages 19–27) participated in the pretest; none of them joined the main experiment. The informed consent was obtained from all the participants. They were instructed to sit on a chair, close their eyes, and spin the fidget spinners handed from an experimenter. Participants were asked to arrange the fidget spinners in order of ease of rotation, with the easiest fidget spinner being rated as 5 and the hardest being rated as 1. The order of the fidget spinners was randomized. The ease of rotation of each fidget spinner was then evaluated by taking the average of the ratings given by the participants.

### Participants

Twenty-four healthy Japanese right-handed adults (six females; age range = 18–34 years) participated in the main experiment. Of all 24 participants, two were excluded due to missing data and errors in the experiment. None of the subjects had a history of neurological or psychiatric disorders. We explained the study and obtained written informed consent from all participants. The study was approved by the Ethics Committee of Doshisha University and was conducted in accordance with the Declaration of Helsinki.

### Procedures

First, the participants were instructed on how to spin the fidget spinner outside of the MRI room. The fidget spinner used in the instruction was a non-magnetic and low-eccentricity one, but which was different from those used in real experiments. Participants were instructed to hold the fidget spinner with their right thumb and middle finger, and to flick it with their left index finger. Then, the participants entered the MRI room, and laid on the MR scanner to conduct the experiment. After the first fixation appeared on the screen for 20 s, participants rotated or held fidget spinners (Fig. 1). In the HOLD condition, when "HOLD" was displayed, they were prepared to flick but did not do it. In the ROTATE condition, participants flicked the spinner with their left index finger when "GO" was displayed, and then kept holding the spinner while the fixation point was shown for 3 s. They repeated this process three times before stopping it when "STOP" was displayed on the screen. The ROTATE and HOLD conditions were repeated alternatively three times each. Both conditions started with the word "READY" shown on the screen. Participants held the spinner with the thumb and middle finger of the right hand and were prepared to flick with their left index finger. Then, the experimenter standing nearby in the MRI room gave the different spinners to the participants and repeated the process until all five types of spinners were tested. Participants were blinded to the spinner type. In addition, participants were asked not to touch or look at the spinner in order to identify its shape. All visual instructions were presented using a presentation software package (Neurobehavioral Systems, Inc., Albany, CA, USA). Following the scan, participants performed a rating test. They rotated each spinner again and orally rated the ease of rotation while lying supine in the MR scanner.

### fMRI acquisition

The T1-weighted structural images (echo time [TE] = 4 ms, repetition time [TR] = 9.4 ms, flip angle = 8, the field of view [FOV] = 256 × 256 mm, 192 slices, inversion time [TI] = 1013 ms, voxel size = 1 × 1 × 1 mm) and echo-planar imaging (EPI) functional images (30 slices; TR = 3000 ms; TE: 50 ms; 4 mm thickness with a 1 mm gap; transverse; FOV: 192 × 192 mm; matrix size: 64 × 64; fractional anisotropy [FA]: 90°) were acquired using a 1.5-T MRI scanner (Echelon Vega, Hitachi Medical, Chiba, Japan) according to a previously reported protocol^33^.

### MRI data analysis

#### Preprocessing

The data were analyzed by SPM12 software (Wellcome Trust Centre for Neuroimaging, UCL Queen Square Institute of Neurology, London, UK; http://www.fil.ion.ucl.ac.uk/spm/). The first five EPI volumes of each run were discarded to allow for signal stabilization. The remaining EPI volumes were realigned, normalized to the Montreal Neurological Institute (MNI) space, and smoothed with a Gaussian kernel of 12-mm full-width-at-half-maximum in the x, y, and z axes.

### Statistical analysis

The analysis of functional imaging data required the creation of statistical parametric maps, which were used to assess hypothesized condition-specific effects using a general linear model. Two types of design matrices were created: one that focused on the effects of conditions (ROTATE and HOLD), and another that considered the effects of both block types (ROTATE and HOLD) and five different types of spinners with varying levels of eccentricity. The regressors were then convolved with a canonical hemodynamic response function, and residual motion effects were modeled by including the realignment parameters. The onset of each regressor was set to coincide with the start of the cues for rotating (i.e., flicking) or holding, and each regressor lasted for 1000 ms. Each component of the model was used as a regressor in a multiple regression analysis for each participant. For each subject, to determine the main effects of rotating spinners, we constructed a contrast image, defined as rotating spinners versus holding spinners [ROTATE − HOLD] using the first design matrix. To identify the main effects of eccentricity, we then looked at a contrast image negatively correlated with eccentricity, defined as [2 × A(ROTATE − HOLD) + B(ROTATE − HOLD) + 0 × C(ROTATE − HOLD) + (− 1) × D(ROTATE − HOLD) + (− 2) × E(ROTATE − HOLD)] using the second matrix. These contrast calculations are based on the hypothesis that there is a difference in brain activity between the ROTATE condition and the HOLD condition, and that there is a gradual change in the amount of activity depending on the type of fidget spinner, with type A having the highest activity and type E having the lowest. Since spinner C did not contribute to the contrast calculation, we also plotted the signal change for each fidget spinner to visually assess the activity level of spinner C.

For a group-level analysis, the participants' contrast maps were subjected to random-effects analyses to consider individual variance. We employed a one sample t-test, with a voxel-level uncorrected threshold of *p* < 0.001 and a cluster-level family-wise error-corrected threshold of P < 0.05. The automated anatomical labeling was used to confirm the regions’ names^34^. MRIcroGL software (https://www.mccauslandcenter.sc.edu/mricrogl/) was used to visualize the fMRI results.

We performed a region of interest (ROI) analysis following the group-level analysis to confirm the gradual change of the BOLD signal in the SMA depending on eccentricity. Each ROI was a sphere with a 4-mm radius and the peak was based on group-level analysis using the second contrast.

### Psycho-physiological interaction analysis

We conducted a PPI analysis to examine the relationship between the SMA and the ventral striatum during rotating or holding fidget spinners. The group-level SMA peak (x = 14, y =  − 8, z = 58) correlating with eccentricity served as the center for the spherical ROI (8-mm radius). The first eigenvariate from this ROI constituted the physiological variable. The psychological variable was the contrast vector representing the task effect of rotating versus holding. These regressors and their interaction terms were estimated at the first level. Contrast images estimated from PPI regressor were then entered into a one-sample t-test. Given the previously hypothesized role of the ventral striatum in rotating fidget spinners, a small volume correction for multiple comparisons (p < 0.05, peak level FWE corrected) was used. For small volume correction, regions of interest were the striatum (the caudate and nucleus accumbens).

## Data Availability

The datasets generated during and analyzed during the current study are available from the corresponding author on reasonable request.
